# Association between herpes simplex virus infection and Alzheimer’s disease biomarkers: analysis within the MAPT trial

**DOI:** 10.1038/s41598-024-84583-x

**Published:** 2025-01-18

**Authors:** Morgane Linard, Isabelle Garrigue, Bruno Vellas, Nicola Coley, Henrik Zetterberg, Kaj Blennow, Nicholas James Ashton, Pierre Payoux, Anne-Sophie Salabert, Jean-François Dartigues, Joachim Mazere, Sandrine Andrieu, Catherine Helmer

**Affiliations:** 1 INSERM U1219 Bordeaux Population Health Research Center, University of Bordeaux, 146, rue Léo Saignat, 33076 Bordeaux Cedex, France; 2 CNRS, MFP, UMR 5234, University of Bordeaux, Bordeaux, France; 3 Virology Department, University Hospital of Bordeaux, Bordeaux, France; 4 Gérontopôle de Toulouse, Institut du Vieillissement, University Hospital of Toulouse, Toulouse, France; 5 INSERM, CERPOP, U1295, University of Toulouse, Toulouse, France; 6 Department of Clinical Epidemiology and Public Health, University Hospital of Toulouse, Toulouse, France; 7 Department of Psychiatry and Neurochemistry, Institute of Neuroscience and Physiology, The Sahlgrenska Academy at the University of Gothenburg, Mölndal, Sweden; 8 Clinical Neurochemistry Laboratory, Sahlgrenska University Hospital, Mölndal, Sweden; 9 Department of Neurodegenerative Disease, UCL Institute of Neurology, Queen Square, London, UK; 10 UK Dementia Research Institute at UCL, London, UK; 11 Hong Kong Center for Neurodegenerative Diseases, Clear Water Bay, Hong Kong, China; 12 Wisconsin Alzheimer’s Disease Research Center, University of Wisconsin School of Medicine and Public Health, University of Wisconsin-Madison, Madison, WI USA; 13 Paris Brain Institute, ICM, Pitié-Salpêtrière Hospital, Sorbonne University, Paris, France; 14 Division of Life Sciences and Medicine, and Department of Neurology, Institute on Aging and Brain Disorders, Neurodegenerative Disorder Research Center, University of Science and Technology of China and First Affiliated Hospital of USTC, Hefei, People’s Republic of China; 15 Institute of Psychiatry, Psychology and Neuroscience, King’s College London, Maurice Wohl Institute Clinical Neuroscience Institute, London, UK; 16 NIHR Biomedical Research Centre for Mental Health and Biomedical Research Unit for Dementia at South London and Maudsley NHS Foundation, London, UK; 17 Centre for Age-Related Medicine, Stavanger University Hospital, Stavanger, Norway; 18 Nuclear Medicine Department, University Hospital of Toulouse, Toulouse, France; 19 INSERM ToNIC, U1214, University of Toulouse, Toulouse, France; 20 Radiopharmacy Department, University Hospital of Toulouse, Toulouse, France; 21 Memory Consultation, CMRR, University Hospital of Bordeaux, Bordeaux, France; 22 Nuclear Medicine Department, University Hospital of Bordeaux, Bordeaux, France; 23 CNRS, INCIA, UMR 5287, University of Bordeaux, Bordeaux, France; 24 INSERM, Bergonié Institute, BPH, U1219, CIC-P 1401, University of Bordeaux, Bordeaux, France

**Keywords:** Virology, Alzheimer's disease, Risk factors

## Abstract

In vitro and animal studies have suggested that inoculation with herpes simplex virus 1 (HSV-1) can lead to amyloid deposits, hyperphosphorylation of tau, and/or neuronal loss. Here, we studied the association between HSV-1 and Alzheimer’s disease biomarkers in humans. Our sample included 182 participants at risk of cognitive decline from the Multidomain Alzheimer Preventive Trial who had HSV-1 plasma serology and an amyloid PET scan. Plasma Aβ42/40 ratio, neurofilament light chain and p-tau181 were also available for a sub-sample of participants. Multivariate linear regressions were performed and stratified by *APOE4* genotype. The median age was 74.0 years, 85.2% were infected with HSV-1. Infected participants tended to have a lower cortical amyloid load than uninfected participants (β = -0.08, p = 0.06), especially those suspected of reactivating HSV-1 most frequently (i.e. with a high anti-HSV-1 IgG level; n = 58, β = -0.09 p = 0.04). After stratification, the association was only significant in *APOE4* carriers (n = 43, β = -0.21 p = 0.01). No association was found with the plasma biomarkers. The trend toward lower cortical amyloid load in HSV-1-infected participants was unexpected given the pre-existing literature and may be explained either by a modified immune response in HSV-1 infected subjects which could favour the clearance of amyloid deposits or by a selection bias.

## Introduction

Different processes underlie the development of Alzheimer’s disease (AD), including intracerebral accumulation of both amyloid β (Aβ) deposits and neurofibrillary tangles (NFTs) (secondary to hyperphosphorylation of the tau protein) and subsequent neuronal loss. The extent of these lesions can be estimated in vivo using neuroimaging methods, including quantification of amyloid and tau lesions by positron emission tomography (PET) scans and measurement of cerebral atrophy by magnetic resonance imaging (MRI)^1^. Well-established cerebrospinal fluid (CSF) biomarkers of these processes also exist. More recently, promising blood-based assays of these biomarkers have also been developed^2–5^, including sensitive and specific assays for the 42 and 40 amino acid-long amyloid β proteins (Aβ42/40 ratio, reduced levels of which reflect Aβ plaque pathology), phosphorylated tau (p-tau, reflecting AD-related tau phosphorylation) and neurofilament light chain (NfL, reflecting neuroaxonal injury irrespective of its cause).

Nevertheless, the triggers of these processes remain insufficiently understood. Among the existing hypotheses, a growing number of studies suggest that inadequate immune control of neurotropic herpes simplex virus 1 (HSV-1) could be involved in the onset of AD^6,7^. In particular, several studies highlighted a link between HSV-1 and AD hallmarks. In vitro, HSV-1 inoculation leads to Aβ accumulation^8–17^, increased concentrations of p-tau and a reduction in neuronal viability^16–21^. In rodents, HSV-1 inoculation leads to intracerebral accumulations of Aβ^9,22^ and p-tau^22,23^ proportional to the number of viral reactivations^22^. Notably, Aβ deposits were found to colocalize with HSV-1 in the brains of mice^24^. Moreover, several studies highlighted that Aβ possesses antimicrobial activity against several viruses, bacteria and fungi^24–26^ and could also play a protective role against various neurotoxic solutes^27^, suggesting that the accumulation of Aβ deposits in the central nervous system (CNS) may be part of the brain’s response to aggression. In humans, only rare studies exist investigating links between HSV infection and plasma amyloid biomarkers^28–30^, plasma NfL^30^, CSF biomarkers^31^, or postmortem intracerebral lesions^32,33^, leading to less convincing results regarding the potential involvement of HSV-1 in AD.

Our primary objective was thus to explore a potential association between HSV-1 and AD biomarkers. We primarily investigated the intracerebral amyloid load measured by PET, for which no study exists to date. We also investigated plasma biomarkers such as the Aβ42/40 ratio, NfL and p-tau181.

## Methods

### Design and participants

The Multidomain Alzheimer Preventive Trial (MAPT, NCT00672685) is a phase 3 multicenter randomized trial^34,35^ that aimed to assess the effect of long-term preventive interventions on cognitive decline in older adults. The 3-year interventions consisting of i) supplementation with omega-3 polyunsaturated fatty acids or ii) multidomain lifestyle interventions (nutritional advice, cognitive training, physical activity) plus placebo or iii) the combination of supplementation and lifestyle interventions. The interventions failed to demonstrate efficacy compared with the placebo group^35^.

Between 2008 and 2011, 1 680 participants at risk of cognitive decline at 13 memory centers in France and Monaco were included in the study according to the following criteria: i) ≥ 70 years old, ii) living in the community and iii) meeting at least one of the following conditions: spontaneous memory complaint expressed to the general practitioner, limitation in one instrumental activity of daily living and walking speed < 0.8 m/s. Participants with dementia, a Mini Mental State Examination (MMSE) < 24, dependency for any basic activity of daily living or a disease compromising the subject’s participation were not included. Each participant provided signed informed consent, and the study protocol was approved by the French Ethical Committee located in Toulouse (CPP SOOM II) and by the French Health Authority. All the methods were performed in accordance with the relevant guidelines and regulations.

**Fig. 1:**
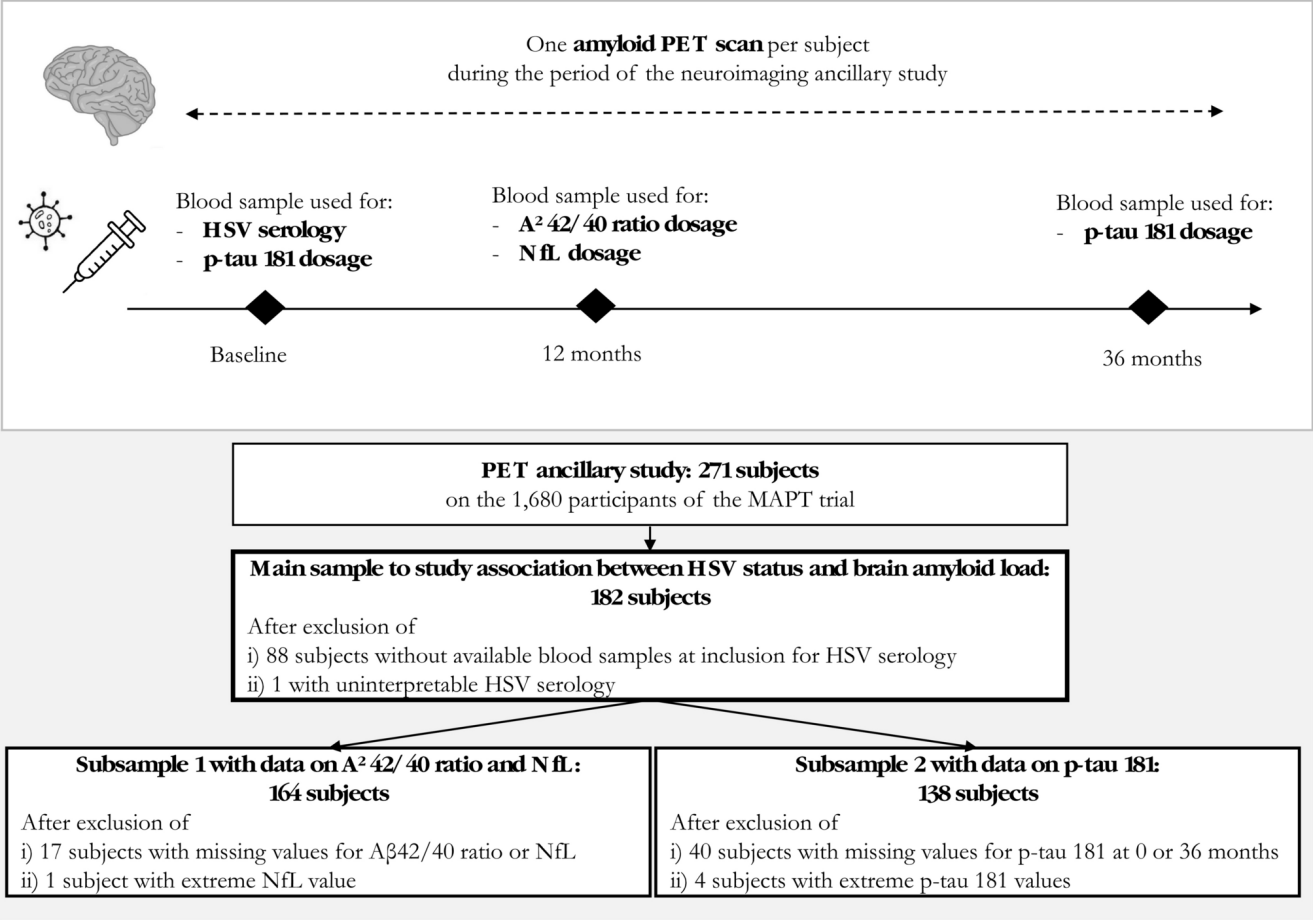
Study design and flow chart. The main sample of our study includes 182 subjects with both HSV-1 serology at baseline and amyloid PET scans that may have been performed throughout follow-up. Two subsamples were used to assess associations between HSV-1 infection and plasma biomarkers: i) a subsample of 164 subjects with dosages of Aβ42/40 ratio and NfL at 12 months and ii) a subsample of 138 subjects with two dosages of p-tau 181 at baseline and 36 months. HSV, herpes simplex virus; NfL, neurofilament light chain; PET, positron emission tomography; p-tau, phosphorylated-tau.

Aiming to explore the associations between HSV-1 infection and biomarkers reflecting pathophysiological processes underlying AD, this secondary analysis included 182 participants with both an amyloid PET scan (performed once during the 3-year follow-up) and an anti-HSV-1 plasma serology (measured at baseline) (Fig. 1). Subsequently, assays of plasma biomarkers were performed in stored blood samples from some of these participants. A first subsample of 164 participants had available data for both the Aβ42/40 ratio and NfL (measured in samples collected at 12 months of follow-up). A second subsample of 138 participants had data for p-tau181 (measured twice, at baseline and 36 months of follow-up).

### Anti-HSV-1 serologies

The presence of anti-HSV-1 immunoglobulin G (IgG) in the blood was assessed using the LIAISON® HSV1 Type Specific IgG kit (details in Supplementary file 1). Of the 271 participants who underwent an amyloid PET scan in the MAPT trial, 183 had an available plasma sample at baseline for performing anti-HSV serologies. One subject was further excluded due to uninterpretable HSV data (Fig. 1).

### Biomarkers related to Alzheimer’s disease

#### Cortical amyloid load

In the MAPT trial, the cortical amyloid load was measured by [18F] florbetapir (AV45) PET^34,36^. Details regarding the inclusion criteria and the acquisition parameters are provided in Supplementary file 1. The timing of the PET was variable and could take place during the 3 years of follow-up of the trial.

A semiautomated quantitative analysis made it possible to estimate regional standard uptake value ratios (SUVR), corresponding to the ratio between the fixation of the tracer in the studied region and that in a reference region (the whole cerebellum in this study). Subsequently, a cortical-to-cerebellar SUVR was defined as the mean signal of six predefined AD-related cortical regions of interest (frontal, temporal, parietal, precuneus, anterior cingulate, and posterior cingulate). Cortical-to-cerebellar SUVR values were analyzed as both continuous and binary variables with an amyloid positivity threshold set at ≥ 1.17^37,38^. Additional analyses were also carried out on separate cortical areas (continuous variable).

#### Plasma biomarkers

AD-related plasma biomarkers were measured using high-precision methods (details in Supplementary file 1)^39–41^. In the first subset, 165 participants had both Aβ42/40 ratios (lower is worse) and NfL (pg/mL, higher is worse) measured at 12 months of follow-up. Among them, one subject with an NfL value four standard deviations above the sample mean was considered an outlier and further excluded. In a second subset, 142 participants had p-tau181 (pg/mL, higher is worse) obtained at both baseline and 36 months of follow-up. Among them, 2 participants were considered outliers, and 2 participants had p-tau181 values below the lower limit of detection (1.0 pg/mL) and were further excluded. In regression models, plasma biomarkers were used as continuous variables, and both NfL and p-tau181 were log-transformed to correct skewness.

#### Statistical analyses

To examine associations between HSV-1 serostatus and AD biomarkers, univariate and multivariate linear regression models (or logistic regression models for the binary PET amyloid positivity variable) were performed on i) the presence of anti-HSV-1 IgG and ii) the level of anti-HSV-1 IgG (considered in terciles due to an upper limit of quantification). A higher level of IgG was interpreted as reflecting a higher frequency of HSV-1 reactivation over time. Analyses were adjusted for baseline age, sex, presence of at least one *APOE4* allele and level of education. Sensitivity analyses were also performed adjusting for factors potentially associated with AD biomarkers but with a low probability of influencing HSV-1 serostatus: history of hypertension, diabetes or dyslipidemia at baseline, time from randomization to PET scan (only for cortical amyloid load), randomization arms (only for biomarkers measured postbaseline), and serum creatinine level at the time of biomarker measurement (for plasma biomarkers only)^5,42,43^. For linear models, normality of residuals and homoscedasticity were assessed using a histogram of residuals, a normal quantile‒quantile plot of residuals and a plot of residuals *versus* predicted values.

Moreover, as suggested by previously published results from postmortem^44^, epidemiological^6,45,46^ or animal studies^47,48^, we assumed that any association between HSV-1 and AD may be modulated by the *APOE4* genotype. Consequently, analyses were stratified by *APOE4* genotype regardless of the significance of the interactions between HSV-1 and APOE4 (see Supplemental Tables for p-values of HSV-1 × APOE4 interactions). In these models, due to the small sample sizes (particularly among *APOE4* carriers), we did not analyze the levels of anti-HSV-1 IgG in terciles.

Finally, given i) the time sequence of amyloid and tau lesions and ii) the suspected antimicrobial role of Aβ^25^, we hypothesized that associations between HSV-1 and p-tau181 may depend on a preexisting amyloid pathology. Thus, analyses on p-tau181 measured at 36 months were also stratified on the cortical amyloid load (< or ≥ 1.17, measured during the follow-up).

All statistical tests were two-tailed, and the threshold for statistical significance was 5%. Analyses were performed with SAS software (version 9.4, SAS Institute).

## Results

In the main sample (Table 1), the median age was 74.0 years, 59.3% of participants were female, and 85.2% of the participants were infected by HSV-1. Participants with a cortical amyloid load above the positivity threshold of 1.17 represented 42.9% of the sample. The characteristics of the two subsamples with plasma biomarkers were broadly similar (Table 2). The median Aβ42/40 ratio was 0.113. As expected, the cortical-to-cerebellar SUVR and Aβ42/40 ratio were negatively correlated. The median NfL level was 76.8 pg/ml, and the median p-tau181 levels were 8.7 pg/ml and 9.1 pg/ml at baseline and 36 months, respectively. The p-tau181 levels were relatively stable between baseline and 36 months (median variation = 0.3 pg/ml). Amyloid-positive participants (according to PET) exhibited higher plasma levels of NfL (88.2 ± 37.3 *versus* 75.2 ± 26.7), p-tau181 at baseline (10.9 ± 3.8 *versus* 8.8 ± 4.2) and p-tau181 at 36 months (11.9 ± 5.1 *versus* 8.3 ± 3.9) than amyloid-negative participants.

**Table 1:** Participant characteristics in the main sample (n = 182). MAPT trial.

		**Total** **(N = 182)** **N (%)**	**Cortical Aβ load < 1.17** **(N = 104)** **N (%)**	**Cortical Aβ load ≥ 1.17** **(N = 78)** **N (%)**
**Characteristics at baseline**				
Age (years)	Median [IQR]	74.0 [71.0–77.0]	74.0 [71.0–77.0]	72.5 [71.0–78.0]
Sex	Woman	108 (59.3)	62 (59.6)	46 (59.0)
Level of education^1^	No diploma or primary school certificate	48 (26.5)	30 (28.8)	18 (23.4)
	Secondary education	50 (27.6)	24 (23.1)	26 (33.8)
	High school diploma or university level	83 (45.9)	50 (48.1)	33 (42.9)
APOE4^1^	At least one allele	43 (25.9)	13 (14.1)	30 (40.5)
Randomization arm	Multidomain plus polyunsaturated fatty acids	49 (26.9)	36 (34.6)	13 (16.7)
	Polyunsaturated fatty acids	43 (23.6)	23 (22.1)	20 (25.6)
	Multidomain plus placebo	40 (22.0)	24 (23.1)	16 (20.5)
	Placebo	50 (27.5)	21 (20.2)	29 (37.2)
MMSE	Median [IQR]	28 [27–29]	29 [28–29]	28 [27–29]
CDR	0	105 (57.7)	62 (59.6)	43 (55.1)
	0.5	77 (42.3)	42 (40.4)	35 (44.9)
Anti-HSV-1 IgG	Positive	155 (85.2)	90 (86.5)	65 (83.3)
**Amyloid PET scan during the follow-up**				
Age at PET scan (years)	Median [IQR]	75.0 |73.0–79.0]	76.0 |73.0–79.0]	74.0 |73.0–80.0]
Time from randomisation to PET scan (months)	< 12 months	51 (28.0)	29 (27.9)	22 (28.2)
	12–24 months	58 (31.9)	36 (34.6)	22 (28.2)
	≥ 24 months	73 (40.1)	39 (37.5)	34 (43.6)
Cortical SUVR	Median [IQR]	1.13 [1.05–1.29]	1.06 [1.02–1.10]	1.33 [1.25–1.43]
Cortical SUVR ≥ 1.17		78 (42.9)	-	-
MMSE at PET scan^2^	Median [IQR]	29 [27–30]	29 |28–30]	28 [27–29]
CDR at PET scan^2^	0	101 (55.5)	57 (54.8)	44 (56.4)
	0.5	79 (43.4)	47 (45.2)	32 (41.0)
	≥ 1	2 (1.1)	0 (0.0)	2 (2.6)

^1^Missing data for the level of education (n = 1) and *APOE4* (n = 16). ^2^At the closest visit to PET scan.

Abbreviations: CDR, Clinical dementia rating; IQR, interquartile range; MMSE, Mini Mental State Examination; PET, positron emission tomography; SUVR, standard uptake value ratio.

**Table 2:** Participant characteristics in subsamples 1 and 2 (n = 164 and 138). MAPT trial.

		**Subsample 1** **Aβ42/40—NfL** **(N = 164)** **N (%)**	**Subsample 2** **p-tau181** **(N = 138)** **N (%)**
**Characteristics at baseline**			
Age (years)	Median [IQR]	73.0 [71.0–77.0]	73.0 [71.0–77.0]
Sex	Woman	97 (59.1)	84 (60.9)
Level of education^1^	No diploma or primary school certificate	41 (25.2)	37 (27.0)
	Secondary education	44 (27.0)	33 (24.1)
	High school diploma or university level	78 (47.9)	67 (48.9)
APOE4^1^	At least one allele	41 (27.2)	33 (26.6)
Randomization arm	Multidomain plus polyunsaturated fatty acids	46 (28.0)	39 (28.3)
	Polyunsaturated fatty acids	38 (23.2)	31 (22.5)
	Multidomain plus placebo	34 (20.7)	27 (19.6)
	Placebo	46 (28.0)	41 (29.7)
MMSE	Median [IQR]	28 [28–29]	28 [28–29]
CDR	0	95 (57.9)	85 (61.6)
	0.5	69 (42.1)	53 (38.4)
Anti-HSV-1 IgG	Positive	139 (84.8)	117 (84.8)
**Plasma biomarkers**			
Aβ42/40 ratio at 12 months	Median [IQR]	0.113 [0.102–0.122]	-
NfL at 12 months (pg/ml)	Median [IQR]	76.8 [58.3–94.0]	-
p-tau181 at baseline (pg/ml)	Median [IQR]	-	8.7 [6.8–12.0]
p-tau181 at 36 months (pg/ml)	Median [IQR]	-	9.1 [6.6–11.9]
Variation of p-tau181 between 0 and 36 months (pg/ml)	Median [IQR]	-	0.3 [-1.4–2.2]
MMSE at 12 or 36 months^2^	Median [IQR]	29 [27–30]	29 [28–29]
CDR at 12 or 36 months^2^	0	87 (53.1)	76 (55.1)
	0.5	75 (45.7)	60 (43.5)
	≥ 1	2 (1.2)	2 (1.4)

^1^Missing data for the level of education (n = 1 for subsample 1 and 1 for subsample 2) and *APOE4* (n = 13 for subsample 1 and 14 for subsample 2). ^2^12 months for subsample 1 and 36 months for subsample 2.

Abbreviations: CDR, Clinical dementia rating; IQR, interquartile range; MMSE, Mini Mental State Examination; NfL, neurofilament light chain; p-tau181, phosphorylated tau 181.

**Fig. 2:**
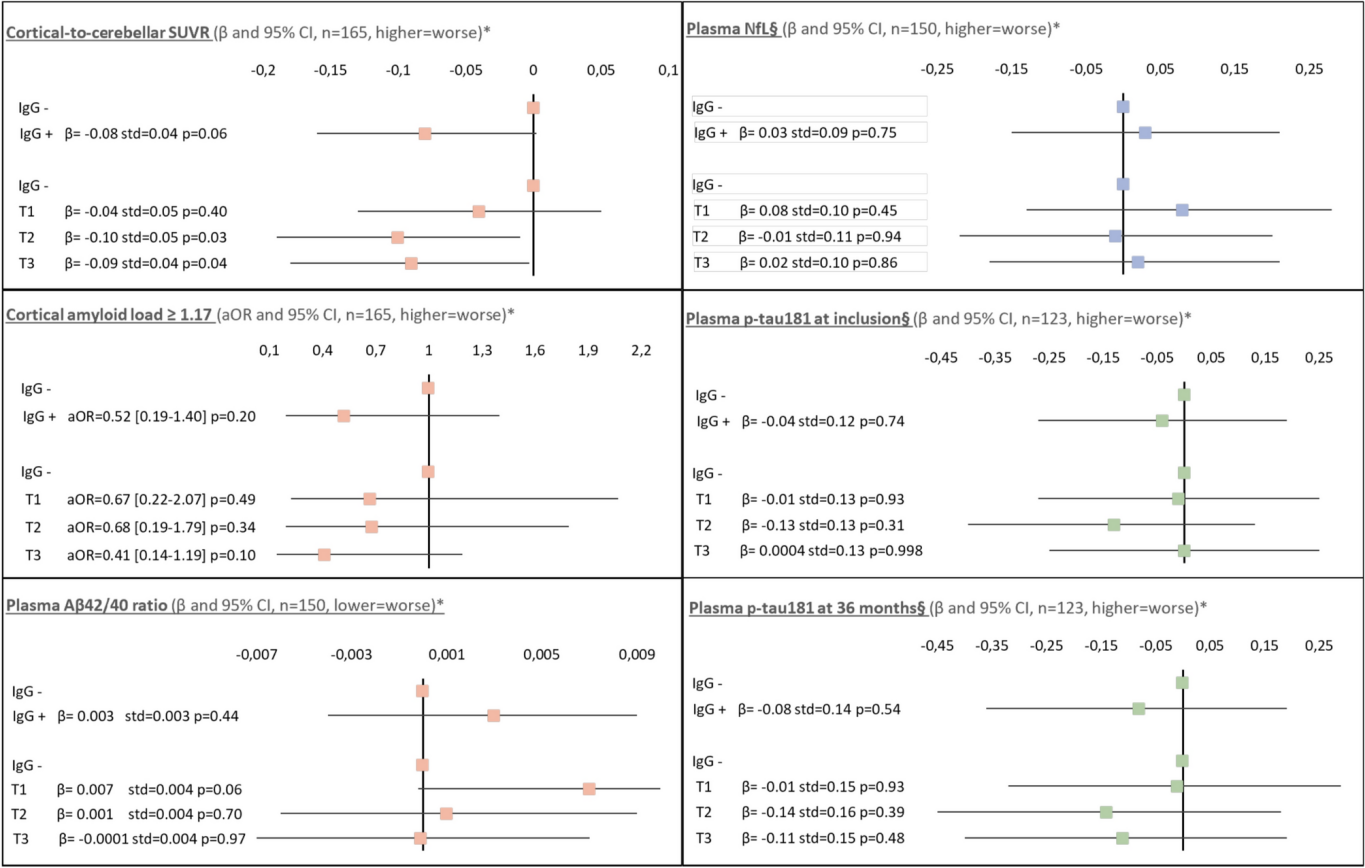
Association between HSV-1 serostatus and Alzheimer’s disease biomarkers: Multivariate regression models. MAPT trial. *To examine associations between HSV-1 serostatus and AD biomarkers, multivariate linear regression models (or logistic regression models for the binary PET amyloid positivity variable) were performed on either i) the presence of anti-HSV-1 IgG (IgG +) or ii) the level of anti-HSV-1 IgG considered in terciles (T1, T2 and T3 corresponding to 1^st^, 2^nd^ and 3^rd^ terciles, respectively). In both cases, the reference category was the absence of anti-HSV-1 IgG (IgG-). Analyses were adjusted for baseline age, sex, presence of at least one *APOE4* allele and level of education. The results are presented in the form of β (and their 95% confidence intervals in the figure), standard deviations and p values for the linear regressions or adjusted odds ratios and their 95% confidence intervals, as well as p values for logistic regressions. ^§^in pg/ml, log transformed. Abbreviations: aOR, adjusted odds ratio; CI, confidence interval; HSV-1, herpes simplex virus 1; IgG, immunoglobulin G; NfL, neurofilament light chain; p-tau181, phosphorylated tau 181; std, standard deviation; SUVR, standard uptake value ratio; T1, T2 and T3, 1^st^, 2^nd^ and 3^rd^ terciles, respectively.

Regarding the associations between anti-HSV1 serology and cortical amyloid load (univariate and multivariate analysis in Supplementary Table S1 and Fig. 2, respectively), participants infected by HSV-1 tended to have a lower cortical SUVR than uninfected participants (β = -0.08, p value = 0.06). Infected participants suspected to reactivate HSV-1 more frequently (those with an anti-HSV-1 IgG level in the 2 highest terciles) also had lower cortical SUVR (β = -0.10, p value = 0.03 for those in the 2^nd^ tercile; β = -0.09, p value = 0.04 for those in the 3^rd^ tercile) than uninfected participants. A similar trend (although not statistically significant) was observed when using the binary PET amyloid positivity variable. Sensitivity analyses (Supplementary Table S1) found similar results. Stratified analyses seem to support a modifying effect of *APOE4* (Supplementary Table S1). Among *APOE4* carriers, the association between being infected by HSV-1 and having a lower cortical amyloid load seemed stronger (n = 43, β = -0.21, p value = 0.01), whereas the association was not statistically significant among *APOE4* noncarriers (n = 122, β = -0.03, p value = 0.45). Complementary analyses carried out on different cortical areas considered separately show similar results (Supplemental Table S2).

Regarding the associations between anti-HSV-1 serology and plasma biomarkers (univariate and multivariate analysis in Supplementary Tables S3-4 and Fig. 2, respectively), no statistically significant association was found between either i) being infected or ii) the level of anti-HSV-1 IgG and any of the plasma biomarkers studied (Aβ42/40 ratio, NfL or p-tau181). Of note, compared with uninfected participants, those with anti-HSV-1 IgG in the first tercile tended to have a higher Aβ42/40 ratio (β = 0.007, p value = 0.06), but there was no significant association for the other terciles of anti-HSV-1 IgG levels. Sensitivity analyses (Supplementary Tables S3-4) found similar results. Analyses stratified on *APOE4* (Supplementary Tables S3-4) did not support the existence of an interaction between HSV-1 and *APOE4* for these biomarkers.

Although far from statistically significant, some results suggest that the existence of an underlying amyloid pathology might modulate the impact of HSV-1 on tau lesions. Indeed, among participants with a cortical amyloid load ≥ 1.17, infected participants tended to have increased levels of p-tau181 at 36 months (β = 0.14, p value = 0.37), whereas among participants with a cortical amyloid load < 1.17, infected participants tended to have decreased levels of p-tau181 (β = -0.24, p value = 0.28).

## Discussion

### Main findings

Regarding the cortical amyloid load measured by PET, our analyses found intriguing results suggesting that participants infected by HSV-1 (and particularly those suspected to reactivate HSV-1 more frequently) tended to have a lower cortical amyloid load than uninfected participants. Moreover, after stratification on the *APOE4* genotype, the association between being infected with HSV-1 and a lower intracerebral amyloid load was only statistically significant in *APOE4* carriers. Conversely, no association was found between either i) being infected by HSV-1 or ii) the level of anti-HSV-1 IgG (suggested to reflect the frequency of viral reactivation) and any of the plasma biomarkers studied (Aβ42/40 ratio, NfL or p-tau181).

### Comparison to previous literature

Our study investigated the association between HSV-1 serostatus and intracerebral amyloid load measured by PET, but previous results exist on intracerebral amyloid load measured postmortem. Olsson et al.^33^ highlighted that among HSV-1-infected participants, 32.4% had intracerebral amyloid deposits compared with 10.5% of uninfected participants, and 39.0% had NFTs compared with 25.0% of uninfected participants. Nevertheless, after adjusting for age, the associations were not statistically significant (p value = 0.2 and 0.8, respectively), and no significant association was found concerning the level of anti-HSV IgG.

Regarding plasma biomarkers, three studies reported results consistent with ours. Féart et al.^28^ found no association between the levels of anti-HSV IgG and the levels of plasma Aβ40, Aβ42 or the Aβ42/40 ratio and no interaction between HSV serostatus and *APOE4*. Note that a significant association was nevertheless found between anti-HSV IgM (marker of recent viral reactivation) and lower levels of plasma Aβ40 or Aβ42 but not with the Aβ42/40 ratio. Lopatko et al.^29^ found no significant association between the presence or levels of plasma anti-HSV-1 IgG and levels of plasma Aβ40 or Aβ42, whether in AD or in healthy participants. Duggan et al.^30^ highlighted no significant association between a history of symptomatic infections with HSV-1, HSV-2 or varicella zoster virus (11.8% of their study sample) and Aβ42/40 ratio or NfL levels and no interaction with *APOE4*.

Recently, Goldhardt et al.^31^ published results on the presence of an intrathecal synthesis of anti-HSV IgG (reflecting viral reactivations within the CNS): the CSF-to-serum antibody index was positively correlated with CSF p-tau and t-tau among AD patients, and this association was modulated by the CSF Aβ42/40 ratio (with stronger associations among participants with low ratios). Notably, in the same study, no association was found between plasma anti-HSV-1 serology and CSF biomarkers.

### Interpretation

Our results suggest that participants infected by HSV-1 (and particularly i) those suspected to reactivate HSV-1 more frequently and ii) *APOE4* carriers) tend to have a lower cortical amyloid load than uninfected participants. This result is unexpected given the various studies showing that HSV-1 inoculation leads to Aβ accumulation in vitro and in animals.

Two possible explanations for these associations should be considered. Firstly, HSV-1 infection may lead to changes in the immune response within the CNS, promoting the clearance of amyloid deposits. Secondly, these results may reflect a selection bias related to the exclusion of subjects with dementia in the MAPT trial. Indeed, under the hypothesis of a role of Aβ in the brain’s defense, diffuse amyloid deposits may accumulate secondary to various attacks of the CNS (including from HSV-1 but not restricted to it). Such a pro-inflammatory environment could in turn trigger HSV-1 reactivation within neurons and, as suggested in vitro and in animals, could lead to subsequent appearance of NFTs whose topographic sequence^49^ seems consistent with HSV-1 tropism for the temporal cortex and its ability to spread to other brain regions following neural networks. (Notably, although far from statistically significant, the fact that among amyloid-positive participants, those infected by HSV-1 had increased levels of p-tau181 at 36 months compared with noninfected participants might argue in favor of this hypothesis.) Overall, if HSV-1 infection promotes progression to dementia only among those with a pre-existing amyloid pathology (while infected subjects with no amyloid pathology would remain asymptomatic), including only participants with no or few objectifiable cognitive symptoms could have led to the selection of infected subjects with fewer underlying amyloid pathology.

The absence of an association between HSV-1 serostatus and any of the plasma biomarkers studied may argue against an AD-promoting effect of HSV-1. Nevertheless, the absence could also be due to insufficient statistical power, the use of plasma biomarkers of AD suffering from large overlaps between groups, or the inclusion of participants at early stages of the disease. Moreover, conversely to the search for an intrathecal synthesis of anti-HSV1 antibodies, plasma serologies do not provide information on the reactivation of HSV-1 within the CNS.

### Limitations

We investigated the association between HSV-1 serostatus and several AD biomarkers, which has been relatively understudied in this context: i) intracerebral amyloid load measured by PET and ii) plasma p-tau181, Aβ42/40 ratio and NfL. However, this approach results in the main limitation of our study: anti-HSV-1 serologies were performed only for participants with an available amyloid PET, thus limiting the statistical power of the analyses.

This study benefited from serologies specific to HSV-1, leading to an estimated seroprevalence close to that usually found in the elderly population^50^. However, plasma serologies are imperfect markers of the frequency of viral reactivations and do not provide information regarding the existence of reactivations within the CNS (unlike intrathecal assays). In addition, while serology was performed at inclusion, amyloid PET scans could have been performed throughout follow-up. If we anticipate a very minimal number of seroconversions between the two measurement points (given that primary HSV-1 infection occurs most of the time in childhood or adolescence), anti-HSV-1 antibody levels could have varied during this period and thus biased the analyses. Nevertheless, given the slow and progressive evolution of amyloid lesions over several years, we anticipate little impact from such variations occurring on the scale of a few months.

With respect to AD biomarkers, florbetapir PET scans provide an accurate estimate of the intracerebral amyloid load^51^. It should be noted that, although a key component of AD, it does not represent a biomarker with good specificity, given that a significant proportion of “amyloid-positive” individuals remain cognitively healthy^1,52^. The plasma Aβ42/40 ratio, although estimated using a high-precision assay based on immunoprecipitation and mass spectrometry methods^39^, suffers from large overlaps between amyloid PET + and PET- participants^53^. Plasma NfL levels were measured using a third generation electrochemiluminescence-based assay. Although less efficient than fourth-generation single molecule array (Simoa) technology^2,54^, this method allows sensitive detection of NfL in the blood^2^, which is well correlated with CSF NfL levels^55^. Nevertheless, increased levels of NfL are not AD-specific and rather reflect the presence of neuroaxonal injury irrespective of its cause^2^. Conversely, plasma p-tau181 has been shown to differentiate AD from other neurodegenerative diseases with good accuracy^56–58^. Levels of plasma p-tau 181 are also increased along the AD continuum^53,58^ (this from the preclinical stage) and exhibit good correlation with CSF levels, tau PET and neuropathology^57,58^.

## Conclusion

Our results suggest that participants infected by HSV-1 (and particularly i) those suspected to reactivate HSV-1 more frequently and ii) *APOE4* carriers) tended to have a lower cortical amyloid load (measured by PET) than uninfected participants. These results are unexpected given the pre-existing literature suggesting accumulation of amyloid deposits secondary to HSV-1 virus inoculation in vivo or in animals. Two possible explanations for these associations should be considered, either a modified immune response in HSV-1 infected subjects promoting the clearance of amyloid deposits or a selection bias related to the exclusion of participants with baseline dementia in the MAPT trial. Moreover, the absence of a statistically significant association between HSV-1 serostatus and any of the plasma biomarkers studied (Aβ42/40 ratio, NfL or p-tau181) may be partly explained by methodological limitations. Overall, discrepancies between these results and those obtained in vitro, in animals or in humans when analyzing intrathecal synthesis of anti-HSV IgG will require additional investigations, especially at different stages of the disease and in larger samples benefiting from CSF samples or PET imaging.

## Supplementary Information

Supplementary Information.

## Data Availability

The datasets used and/or analysed during the current study are available from the MAPT data sharing committee on reasonable request (nicola.coley@inserm.fr).
