# Supplementary material for: Association between herpes simplex virus infection and Alzheimer’s disease biomarkers: analysis within the MAPT trial
Source: Sci Rep. 2025 Jan 18;15:2362. doi: 10.1038/s41598-024-84583-x (PMC11748617; doi:10.1038/s41598-024-84583-x)
Supplement: Supplementary file 1 — Supplementary Information. [file 41598_2024_84583_MOESM1_ESM.pdf]

Association between herpes simplex virus infection and Alzheimer's disease biomarkers: analysis within the MAPT trial

Authors: Morgane LINARD[1*] MD PhD, Isabelle GARRIGUE[2,3] MD PhD, Bruno VELLAS[4,5] MD PhD, Nicola COLEY[5,6] PhD, Henrik ZETTERBERG[7-12] MD PhD, Kaj BLENNOW[7-8,13-14] MD PhD, Nicholas James ASHTON[7,15-17] PhD, Pierre PAYOUX[18-19] MD PhD, Anne-Sophie SALABERT[19-20] PharmD PhD, Jean-François DARTIGUES[1,21] MD PhD, Joachim MAZERE[22,23] PharmD PhD, Sandrine ANDRIEU[5,6] MD PhD, Catherine HELMER[24] MD PhD

Affiliations:
[1]University of Bordeaux, INSERM, BPH, U1219, Bordeaux, France
[2]University of Bordeaux, CNRS, MFP, UMR 5234, Bordeaux, France
[3]University Hospital of Bordeaux, Virology department, Bordeaux, France
[4]University Hospital of Toulouse, Gérontopôle de Toulouse, Institut du Vieillissement, Toulouse, France
[5]University of Toulouse, INSERM, CERPOP, U1295, Toulouse, France
[6]University Hospital of Toulouse, Department of Clinical Epidemiology and Public Health, Toulouse, France
[7]Department of Psychiatry and Neurochemistry, Institute of Neuroscience and Physiology, the Sahlgrenska Academy at the University of Gothenburg, Mölndal, Sweden
[8]Clinical Neurochemistry Laboratory, Sahlgrenska University Hospital, Mölndal, Sweden
[9]Department of Neurodegenerative Disease, UCL Institute of Neurology, Queen Square, London, UK
[10]UK Dementia Research Institute at UCL, London, UK
[11]Hong Kong Center for Neurodegenerative Diseases, Clear Water Bay, Hong Kong, China
[12]Wisconsin Alzheimer's Disease Research Center, University of Wisconsin School of Medicine and Public Health, University of Wisconsin-Madison, Madison, WI, USA
[13]Paris Brain Institute, ICM, Pitié-Salpêtrière Hospital, Sorbonne University, Paris, France
[14]Neurodegenerative Disorder Research Center, Division of Life Sciences and Medicine, and Department of Neurology, Institute on Aging and Brain Disorders, University of Science and Technology of China and First Affiliated Hospital of USTC, Hefei, P.R. China
[15]King's College London, Institute of Psychiatry, Psychology and Neuroscience Maurice Wohl Institute Clinical Neuroscience Institute, London, UK
[16]NIHR Biomedical Research Centre for Mental Health and Biomedical Research Unit for Dementia at South London and Maudsley NHS Foundation, London, UK
[17]Centre for Age-Related Medicine, Stavanger University Hospital, Stavanger, Norway.
[18]University Hospital of Toulouse, Nuclear medicine department, Toulouse France
[19]University of Toulouse, INSERM ToNIC, U1214, Toulouse, France
[20]University Hospital of Toulouse, Radiopharmacy department, Toulouse, France
[21]University Hospital of Bordeaux, Memory consultation, CMRR, Bordeaux, France
[22]University Hospital of Bordeaux, Nuclear medicine department, Bordeaux, France
[23]University of Bordeaux, CNRS, INCIA, UMR 5287, Bordeaux, France
[24]University of Bordeaux, INSERM, Bergonié Institute, BPH, U1219, CIC-P 1401, Bordeaux, France

Supplementary files

a. Supplementary file 1 – Methodological details

Anti-HSV serology

The presence of anti-HSV-1 immunoglobulin G (IgG) in the blood was assessed using the LIAISON® HSV1 Type Specific IgG kit (Diasorin – Italy – chemiluminescence immunoassay technology). The presence of anti-HSV-1/2 immunoglobulin M (IgM), reflecting either a primary infection or a recent reactivation, was assessed using the LIAISON® HSV1/2 IgM kit. As recommended by the manufacturers, i) an index value ≥1.10 was considered to indicate the presence of IgG (or IgM), ii) an index value < 0.9 was considered to indicate the absence of IgG (or IgM) and iii) no conclusion could be made for index values between 0.9 and 1.10. In the latter case, a second serology was performed to verify the result.

Of the 271 participants who underwent an amyloid PET scan in the MAPT trial, 183 participants had an available plasma sample at baseline for performing anti-HSV serologies. One subject was further excluded from the analysis because he was negative for anti-HSV-1 IgG but positive for anti-HSV-1/2 IgM (reflecting either measurement errors or a primary infection, which is relatively unlikely given his age).

Notably, the low prevalence of IgM-positive participants in the sample (1.1% - n=2) prevented the study of this marker.

Amyloid PET scans

Details regarding the inclusion criteria of the amyloid PET ancillary study and acquisition parameters were described previously [1–3]. Briefly, the ancillary study started in July 2010 (after obtaining funding) and was proposed to participants enrolled in the ten

centers close to one of the five nuclear medicine departments offering amyloid PET scans (Bordeaux, Limoges, Montpellier, Nice and Toulouse). The protocol was approved by the French Ethical Committee located in Toulouse in December 2007 and included participants who signed an additional consent form. [18F] Florbetapir (AV45) PET scans were performed on five different hybrid PET-CT scanners, including one PET CT 690 (GE Healthcare; Cleveland, OH), one Discovery RX VCT (General Electric; Fairfield, CT), two True Point HiRez (Siemens Medical Solutions; Malvern, PA), and one Biograph 4 Emission Duo LSO (Siemens Medical Solutions). All scanners were operated in 3D detection mode. PET sinograms were reconstructed with a 3D iterative algorithm, with corrections for randomness, scatter, photon attenuation and decay, which produced images with an isotropic voxel of 2 × 2 × 2 mm and a spatial resolution of approximately 5-mm full-width at a half-maximum at the center of the field of view. The acquisition data were processed using the standard package delivered with each acquisition system. Data acquisition began 50 min after injection of a mean of 4 MBq/kg weight of [18F] florbetapir. In each subject, 10-min or 15-min frames were acquired to ensure movement-free image acquisition. For the semiautomated quantitative analysis, the 18F-AV45 PET images were coregistered to the 18F-AV45 template provided by Avid Radiopharmaceuticals. A quality control based on a semiquantification process was also provided by Avid.

Plasma biomarkers

The plasma Aβ42/40 ratio and NfL concentration were measured in blood samples taken at the 12-month follow-up (on the same day as the cognitive tests) and stored in EDTA-coated tubes.

The plasma Aβ42/40 ratio was measured using immunoprecipitation and mass spectrometry, as previously described [4,5]. Prior to immunoprecipitation, analytical internal standards were determined by spiking samples with a known quantity of 12C15N-Aβ40 and 12C15N-Aβ42.

Aβ42 and Aβ40 isoforms were then simultaneously immunoprecipitated from 0.45 mL of plasma via a monoclonal anti-Aβ mid-domain antibody (HJ5.1, anti-Aβ13-28) conjugated to M-270 Epoxy Dynabeads (Invitrogen, Waltham, Massachusetts, USA). Protein digestion into peptides was performed using LysN endoprotease (Pierce, Thermo Fisher Scientific, Waltham, Massachusetts, USA). Liquid chromatography–mass spectrometry was performed as described previously [6]. An Orbitrap Fusion Lumos Tribrid mass spectrometer (Thermo Fisher, Waltham, MA) interfaced with an M-class nanoAcquity chromatography system (Waters Corporation, Milford, Massachusetts, USA) was used to analyze plasma for targeted parallel reaction monitoring. For the analysis of Aβ isoforms, the precursor and product ion pairs were chosen as previously described [7,8]. Derived integrated peak areas were analyzed using the Skyline software package [9]. Aβ42 and Aβ40 concentrations (in pg/ml) were calculated by integrated peak area ratios to known concentrations of the internal standards. The plasma Aβ42/40 ratio was then determined by dividing Aβ42 by Aβ40.

The plasma NfL concentration (pg/ml) was measured by an electrochemiluminescence-based assay using the R-PLEX human neurofilament L antibody set (Meso Scale Discovery, F217X-3) with MSD Gold 96-well Small Spot SA SECTOR plates (L45A-1) [4,10]. Samples were diluted twice in Diluent 12 (R50JA-3) and assayed in duplicate following the manufacturer's instructions. The mean value of the duplicate assay measurements was used for analyses.

The plasma p-tau181 concentration (pg/ml) was measured in blood samples obtained at baseline and at 36 months of follow-up. The assays were carried out in the Clinical Neurochemistry Laboratory, University of Gothenburg (Mölndal, Sweden) following an in-house Simoa method previously described in detail [11]. Briefly, the AT270 mouse monoclonal antibody (MN1050, Invitrogen), which recognizes the tau sequence phosphorylated specifically at threonine 181, was coupled to paramagnetic beads (103207; Quanterix) and used for capture. Conjugated to biotin (A3959; Thermo Scientific), the anti-tau mouse monoclonal antibody tau12 (806502, BioLegend), which binds the N-terminal epitope 6-18 on

human tau protein, was used as a detector. Full-length recombinant tau-441 phosphorylated by glycogen synthase kinase 3β (TO8–50FN, SignalChem) was used as a calibrator.

References

1. Payoux, P. *et al.* Cognitive and functional patterns of nondemented subjects with equivocal visual amyloid PET findings. *Eur. J. Nucl. Med. Mol. Imaging* **42**, 1459–1468 (2015).
2. Lilamand, M. *et al.* Brain Amyloid Deposition Is Associated With Lower Instrumental Activities of Daily Living Abilities in Older Adults. Results From the MAPT Study. *J. Gerontol. A. Biol. Sci. Med. Sci.* **71**, 391–397 (2016).
3. Del Campo, N. *et al.* Relationship of regional brain β-amyloid to gait speed. *Neurology* **86**, 36–43 (2016).
4. Raffin, J. *et al.* Associations Between Physical Activity, Blood-Based Biomarkers of Neurodegeneration, and Cognition in Healthy Older Adults: The MAPT Study. *J. Gerontol. A. Biol. Sci. Med. Sci.* **76**, 1382–1390 (2021).
5. Lu, W.-H. *et al.* Investigating the combination of plasma amyloid-beta and geroscience biomarkers on the incidence of clinically meaningful cognitive decline in older adults. *GeroScience* **44**, 1489–1503 (2022).
6. Schindler, S. E. *et al.* High-precision plasma β-amyloid 42/40 predicts current and future brain amyloidosis. *Neurology* **93**, e1647–e1659 (2019).
7. Ovod, V. *et al.* Amyloid β concentrations and stable isotope labeling kinetics of human plasma specific to central nervous system amyloidosis. *Alzheimers Dement. J. Alzheimers Assoc.* **13**, 841–849 (2017).
8. Mawuenyega, K. G., Kasten, T., Sigurdson, W. & Bateman, R. J. Amyloid-beta isoform metabolism quantitation by stable isotope-labeled kinetics. *Anal. Biochem.* **440**, 56–62 (2013).
9. Pino, L. K. *et al.* The Skyline ecosystem: Informatics for quantitative mass spectrometry proteomics. *Mass Spectrom. Rev.* **39**, 229–244 (2020).
10. He, L. *et al.* Plasma neurofilament light chain is associated with cognitive decline in non-dementia older adults. *Sci. Rep.* **11**, 13394 (2021).
11. Karikari, T. K. *et al.* Blood phosphorylated tau 181 as a biomarker for Alzheimer’s disease: a diagnostic performance and prediction modelling study using data from four prospective cohorts. *Lancet Neurol.* **19**, 422–433 (2020).

b. Supplementary table S1: Association between HSV-1 serostatus and cortical amyloid load. Univariate, sensitivity and stratified models.

	Univariate analysis n= 165			Sensitivity analysis[1] n= 165			Interact. APOE4	Among *APOE4* carriers[2] n= 43			Among *APOE4* non carriers[2] n= 122		
**Cortical-to-cerebellar SUVR**	Beta	Std	P value	Beta	Std	P value	P value	Beta	Std	P value	Beta	Std	P value
Anti-HSV-1 IgG													
Positive (vs negative)	- 0.08	0.04	0.08	- 0.07	0.04	0.08	0.08	- 0.21	0.08	0.01	- 0.03	0.05	0.45
Anti-HSV-1 IgG level													
1st tercile (vs negative)	- 0.03	0.05	0.50	- 0.04	0.05	0.43							
2nd tercile (vs negative)	- 0.09	0.05	0.06	- 0.10	0.05	0.03							
3rd tercile (vs negative)	- 0.10	0.05	0.04	- 0.08	0.04	0.07							
**Cortical amyloid load ≥ 1.17**	OR	95% CI	P value	aOR	95% CI	P value	P value	Beta	Std	P value	Beta	Std	P value
Anti-HSV-1 IgG													
Positive (vs negative)	0.55	0.22-1.39	0.21	0.51	0.18-1.42	0.20							
Anti-HSV-1 IgG level													
1st tercile (vs negative)	0.72	0.25-2.05	0.53	0.64	0.20-2.04	0.45							
2nd tercile (vs negative)	0.65	0.23-1.87	0.43	0.57	0.17-1.89	0.36							
3rd tercile (vs negative)	0.40	0.14-1.10	0.07	0.41	0.14-1.25	0.12							

[1] Adjusted for age, sex, *APOE4* genotype, education, history of hypertension, diabetes or dyslipidemia at baseline, randomization arm and time from randomisation to PET scan

[2] Adjusted for age, sex and education. In these models, due to the small sample sizes (particularly among *APOE4* carriers), we did not consider the levels of IgG in terciles. Analyses using the binary variable "cortical amyloid load ≥ 1.17" were not performed due to the absence of uninfected participants with a cortical amyloid load ≥ 1.17 among *APOE4* carriers.

Abbreviations: AD, Alzheimer's disease; aOR, adjusted odds ratio; CI, confidence interval; HSV-1, herpes simplex virus 1; IgG, immunoglobulin G; OR, odds ratio; std, standard deviation; SUVR, standard uptake value ratio.

c. Supplementary table S2: Association between HSV-1 serostatus and cortical amyloid load in several brain areas. Multivariate and stratified models.

SUVR in several brain areas	Multivariate analysis[1] n= 165				Among APOE4 carriers[2] n= 43			Among APOE4 non carriers[2] n= 122		
	Beta	Std	P value	Interaction APOE4	Beta	Std	P value	Beta	Std	P value
**Anterior cingulate cortex**										
Anti-HSV-1 IgG										
Positive (vs negative)	-0.06	0.05	0.22	**0.006**	**-0.32**	**0.10**	**0.004**	0.02	0.06	0.68
Anti-HSV-1 IgG level										
1st tercile (vs negative)	0.003	0.06	0.96							
2nd tercile (vs negative)	-0.09	0.06	0.13							
3rd tercile (vs negative)	-0.09	0.06	0.09							
**Posterior cingulate cortex**										
Anti-HSV-1 IgG										
Positive (vs negative)	**-0.09**	**0.04**	**0.04**	0.32	-0.17	0.10	0.08	-0.06	0.05	0.20
Anti-HSV-1 IgG level										
1st tercile (vs negative)	-0.05	0.05	0.31							
2nd tercile (vs negative)	**-0.11**	**0.05**	**0.02**							
3rd tercile (vs negative)	**-0.10**	**0.05**	**0.04**							
**Precuneus**										
Anti-HSV-1 IgG										
Positive (vs negative)	-0.10	0.06	0.08	0.10	**-0.28**	**0.12**	**0.03**	-0.04	0.06	0.50
Anti-HSV-1 IgG level										
1st tercile (vs negative)	-0.06	0.06	0.37							
2nd tercile (vs negative)	**-0.13**	**0.06**	**0.04**							
3rd tercile (vs negative)	-0.10	0.06	0.09							
**Temporal cortex**										
Anti-HSV-1 IgG										
Positive (vs negative)	**-0.09**	**0.04**	**0.02**	0.15	**-0.20**	**0.07**	**0.01**	-0.05	0.04	0.23
Anti-HSV-1 IgG level										
1st tercile (vs negative)	-0.05	0.04	0.26							
2nd tercile (vs negative)	**-0.11**	**0.04**	**0.01**							
3rd tercile (vs negative)	**-0.10**	**0.04**	**0.02**							
**Medial occipito-frontal cortex**										
Anti-HSV-1 IgG										
Positive (vs negative)	-0.06	0.04	0.17	**0.03**	**-0.23**	**0.08**	**0.008**	-0.002	0.05	0.97
Anti-HSV-1 IgG level										
1st tercile (vs negative)	-0.01	0.05	0.91							
2nd tercile (vs negative)	-0.09	0.05	0.07							
3rd tercile (vs negative)	-0.08	0.05	0.09							
**Medial orbito-frontal cortex**										
Anti-HSV-1 IgG										
Positive (vs negative)	-0.05	0.03	0.09	0.63	-0.10	0.06	0.09	-0.04	0.04	0.24
Anti-HSV-1 IgG level										
1st tercile (vs negative)	-0.04	0.04	0.33							
2nd tercile (vs negative)	-0.06	0.04	0.09							
3rd tercile (vs negative)	-0.06	0.04	0.07							
**Fronto-parietal cortex**										
Anti-HSV-1 IgG										
Positive (vs negative)	-0.08	0.04	0.07	0.08	**-0.22**	**0.08**	**0.01**	-0.03	0.05	0.52
Anti-HSV-1 IgG level										
1st tercile (vs negative)	-0.05	0.05	0.35							
2nd tercile (vs negative)	**-0.10**	**0.05**	**0.03**							
3rd tercile (vs negative)	-0.08	0.05	0.09							

[1] Adjusted for age, sex, APOE4 genotype and education.

[2] Adjusted for age, sex and education. In these models, due to the small sample sizes (particularly among APOE4 carriers), we did not consider the levels of IgG in terciles.

[illegible]

uptake value ratio

d. Supplementary table S3: Association between HSV-1 serostatus and plasma Aβ42/40 ratio or NfL. Univariate, sensitivity and stratified models.

	Univariate analysis n=150			Sensitivity analysis[1] n=142			Interaction APOE4	Among *APOE4* carriers[2] n= 41			Among *APOE4* non carriers[2] n= 109		
**Plasma Aβ42/40 ratio**	Beta	Std	P value	Beta	Std	P value	P value	Beta	Std	P value	Beta	Std	P value
Anti-HSV-1 IgG													
Positive (vs negative)	0.003	0.003	0.43	0.001	0.003	0.75	0.64	0.002	0.006	0.67	0.004	0.004	0.36
Anti-HSV-1 IgG level													
1st tercile (vs negative)	0.007	0.004	0.05	0.006	0.004	0.11							
2nd tercile (vs negative)	0.001	0.004	0.87	0.0004	0.004	0.91							
3rd tercile (vs negative)	0.001	0.004	0.89	- 0.002	0.003	0.60							
**Plasma NfL[3]**	Beta	Std	P value	Beta	Std	P value	P value	Beta	Std	P value	Beta	Std	P value
Anti-HSV-1 IgG													
Positive (vs negative)	0.004	0.09	0.96	0.03	0.09	0.70	0.72	0.06	0.19	0.76	0.009	0.10	0.94
Anti-HSV-1 IgG level													
1st tercile (vs negative)	0.05	0.11	0.64	0.08	0.10	0.42							
2nd tercile (vs negative)	- 0.04	0.11	0.70	0.02	0.11	0.84							
3rd tercile (vs negative)	0.002	0.10	0.99	0.01	0.10	0.93							

[1] Adjusted for age, sex, *APOE4* genotype, education, history of hypertension, diabetes or dyslipidemia at baseline, randomisation arms and serum creatinine levels at 12 months

[2] Adjusted for age, sex and education. In these models, due to the small sample sizes (particularly among *APOE4* carriers), we did not consider the levels of IgG in terciles.

[3] in pg/ml, log-transformed

Abbreviations: AD, Alzheimer's disease; HSV-1, herpes simplex virus 1; IgG, immunoglobulin G; NfL, neurofilament light chain; std, standard deviation.

e. **<u>Supplementary table S4: Association between HSV-1 serostatus and plasma p-tau181. Univariate, sensitivity and stratified models.</u>**

	**Univariate analysis n= 123**			**Sensitivity analysis[1] n= 110 or 118**			**Interaction APOE4**	**Multivariate analysis among *APOE4* carriers[2] n= 33**			**Multivariate analysis among *APOE4* non carriers[2] n= 90**		
**Plasma p-tau181 at baseline[3]**	Beta	Std	P value	Beta	Std	P value	P value	Beta	Std	P value	Beta	Std	P value
Anti-HSV-1 IgG													
Positive (vs negative)	- 0.004	0.12	0.97	0.004	0.12	0.98	0.81	- 0.15	0.22	0.50	- 0.03	0.14	0.83
Anti-HSV-1 IgG level													
1st tercile (vs negative)	0.02	0.13	0.85	0.03	0.14	0.82							
2nd tercile (vs negative)	- 0.08	0.13	0.53	- 0.13	0.14	0.37							
3rd tercile (vs negative)	0.04	0.13	0.78	0.07	0.14	0.60							
**Plasma p-tau181 at 36 months[3]**	Beta	Std	P value	Beta	Std	P value	P value	Beta	Std	P value	Beta	Std	P value
Anti-HSV-1 IgG													
Positive (vs negative)	-0.06	0.14	0.66	- 0.09	0.15	0.54	0.65	- 0.06	0.21	0.77	- 0.12	0.18	0.49
Anti-HSV-1 IgG level													
1st tercile (vs negative)	0.04	0.15	0.82	- 0.04	0.17	0.80							
2nd tercile (vs negative)	- 0.11	0.16	0.49	- 0.13	0.17	0.47							
3rd tercile (vs negative)	- 0.10	0.15	0.50	- 0.10	0.16	0.52							

[1] Adjusted for age, sex, *APOE4* genotype, education, history of hypertension, diabetes or dyslipidemia at baseline and serum creatinine levels at the time of measurement + randomisation arms for p-tau181 at 36 months. Numbers for p-tau181 at baseline and at 36 months, respectively.
[2] Adjusted for age, sex and education. In these models, due to the small sample sizes (particularly among *APOE4* carriers), we did not consider the levels of IgG in terciles.
[3] in pg/ml, log-transformed
Abbreviations: AD, Alzheimer's disease; HSV-1, herpes simplex virus 1; IgG, immunoglobulin G; ptau-181, phosphorylated tau 181; std, standard deviation
